# Comprehensive Analysis Reveals Two Distinct Evolution Patterns of *Salmonella* Flagellin Gene Clusters

**DOI:** 10.3389/fmicb.2017.02604

**Published:** 2017-12-22

**Authors:** Yue Liu, Dao-Feng Zhang, Xiujuan Zhou, Li Xu, Lida Zhang, Xianming Shi

**Affiliations:** MOST-USDA Joint Research Center for Food Safety, School of Agriculture and Biology, and State Key Laboratory of Microbial Metabolism, Shanghai Jiao Tong University, Shanghai, China

**Keywords:** *Salmonella enterica*, flagellin genes, *fliC*, *fljB*, recombination

## Abstract

*Salmonella* is one of the primary causes of foodborne disease, especially *Salmonella enterica* subsp. *enterica* (I) which has caused ~99% of clinical salmonellosis cases for humans and domestic mammals. The flagella genes, *fliC* and *fljB*, which encode the *Salmonella* phase 1 and phase 2 antigens respectively, are considered as the *Salmonella* serotype determinant genes, and contribute to the virulence of *Salmonella*. However, the evolution of the two flagellin genes is still not well-understood. In this study, the *fliC* and *fljB* gene clusters were analyzed among 205 *S. enterica* subspecies I genomes. The dataset covered 87 different serovars of *S. enterica* subsp. *enterica* and included 9 genomes (six serovars) of four other *Salmonella* subspecies. Based on a pan-genome definition and flanked gene linkages, the *fliC* and *fljB* gene clusters were identified in 207 (91 serovars) and 138 (61 serovars) genomes, respectively. A phylogenetic tree constructed based on SNPs (Single Nucleotide Polymorphisms) of core genes were used to reflect the essential evolutionary relationships among various serovars. Congruence analysis was performed among the core genome and each gene of *fliC* and *fljB* gene clusters, with only *fliA* and *fliS* showing congruence to *Salmonella* core genome. Congruence was also observed among *fliB, fliC/fljB*, and *fliD* genes, and their phylogeny revealed a division into two major groups, which strongly corresponded to monophasic and biphasic serovars. Besides, homologous recombination events referring *fliB, fliC*, and *fliD* were found to have mainly occurred within each group. These results suggested two distinct evolutionary patterns of *Salmonella* flagellin gene clusters. Further insight on the evolutionary implication of the two patterns and a framework for phase variation mechanism are needed to be further processed.

## Introduction

*Salmonella* is one of the primary causes of foodborne illness around the world, leading to a huge burden of illness calculated at nearly 93.8 million global cases annually (Majowicz et al., 2010). For understanding the epidemiology of these microorganisms and the investigation of outbreaks caused by *Salmonella*, serotyping has been greatly facilitated by the characterization of isolates. The *Salmonella* genus is composed of two species, *Salmonella bongori* and *Salmonella enterica*, the latter being further classified into six subspecies, *enterica* (I), *salamae* (II), *arizonae* (IIIa), *diarizonae* (IIIb), *houtenae* (IV), and *indica* (VI) (Grimont and Weill, 2007). *Salmonella* is an extremely diverse species, of which more than 2,500 serovars has been reported. *S. enterica* subsp. *enterica* (I) strains, the vast majority of *Salmonella* strains, could be isolated from humans and warm-blooded animals, while all the other subspecies and *S. bongori* are more frequently isolated from cold-blooded animals (Nataro et al., 2011). Importantly, *S. enterica* subsp. *enterica* (I) has caused ~99% of diagnosed *salmonellosis* cases for humans and domestic mammals (Centers for Disease Control and Prevention (CDC), 2011). Strains with different serotypes show different levels of variation, a single host (e.g., *S*. Typhi) being adapted by monomorphic serotypes comprise a single strain in a single lineage, whilst polymorphic serotypes comprise multiple lineages of strains which may or may not be host adapted (e.g., *S*. Choleraesuis) (Wain and O'Grady, 2017).

Based on White-Kauffmann scheme, the *Salmonella* strains have been identified by their surface antigens, on the basis of somatic (O), flagellar (H), and capsular (Vi) antigens (Brenner et al., 2000) and their reactivity to antisera (Schrader et al., 2008). Flagella (*fliC* or *fljB*) protein is the primary globular protein forming the filament of flagellum in the majority of *Salmonella* strains, which functions as a main structural subunit (Haiko and Westerlund-Wikstrom, 2013). These two flagellin genes in most *Salmonella* strains, *fliC* and *fljB*, encode the phase 1 and phase 2 flagellins, respectively. The *fliC* gene is existent in all *Salmonella* cells and has homologs in other enteric bacteria, located in one of the flagellar biosynthesis operons (Macnab, 1992). The *fljB* gene is unique to *S. enterica* and is present in four of the *S. enterica* subspecies (subspecies I, II, IIIb, and VI), separately located on the chromosome from the *fliC* gene (McQuiston et al., 2004). However, these two flagellin genes cannot be expressed simultaneously. The second flagellin locus is considered as a genetic “spare tyre” availed in certain environmental circumstances (McQuiston et al., 2008). *Salmonella* isolates are indicated biphasic, monophasic, or non-motile according to the “phase variation,” expressing both, one, or no flagellar phases, which is regulated by a switch mechanism, an invertible element *hin* (Bonifield and Hughes, 2003). The *fljB* is considered as a duplication of *fliC* but is flanked with the genes, *fljA* and *hin* (Szekely and Simon, 1983), involving in the switch mechanism, which appears to be specific to *Salmonella* (Zieg et al., 1978). Recombination of these genes is the basic source of flagella antigenic variation (Smith et al., 1990) and may occur between *fliC* of co-infecting organisms or *fliC* and *fljB* of the same isolate (Okazaki et al., 1993). The function of flagellar genes have been studied well; the *fliA* existing upstream of the coding region is specific to promoters for flagellar operons (Ohnishi et al., 1990), *fliB* is responsible for flagellar methylation (Parkhill et al., 2001), *fliC* encodes the flagellar filament protein (Ogushi et al., 2001), and *fliD* encodes the filament cap protein of the flagellar apparatus (Yokoseki et al., 1995). The expression of *fliS* facilitates the export of flagellin through the flagellum-specific export pathway (Yokoseki et al., 1995), whereas the *fljB* gene, which expresses phase II flagellin protein, constitutes an operon with the *fljA* gene, which encodes a negative regulator for *fliC* expression (Yamamoto and Kutsukake, 2006).

The flagellin genes are useful as a model system for studying genetic differentiation (Okazaki et al., 1993). Previous study has revealed that the 5′ and 3′ ends of the *fliC* and *fljB* gene sequences from different antigenic types are highly conserved, but the central region sequences can be highly variable (Okazaki et al., 1993). A molecular serotyping for the H antigens by targeting the genes encoding the immunologically recognized antigen could be linked to traditional serotyping methods (McQuiston et al., 2011). Based on phylogenetic relationships adapted from previous studies, the acquisition of phase variation, the biphasic *Salmonella* flourished (McQuiston et al., 2008). However, this conclusion was drawn from analysis of limited numbers of samples and the basic evolutionary process of this apparently high level of surface-antigen diversity in the *Salmonella* is not well-understood (McQuiston et al., 2008). There is also a lack of data on the detailed phylogenetic description of the flagellar gene relationship among the *Salmonella*, as a framework for evolutionary studies. Therefore, it is worthwhile to seek full evolutionary insights and the evolutionary relationships of the *Salmonella* flagellin alleles to estimate the recombination within these loci. So, in this study, comparative genomics was performed to obtain additional evolutionary information regarding flagellin genes, which showed that there are two distinct evolutionary patterns of *fliC* and *fljB* clusters during the speciation and serovar diversification of *Salmonella*.

## Materials and methods

### Data collection

A total of 214 *Salmonella* complete genomes and genome assemblies of highest quality (Table S1) were retrieved from the NCBI (National Center for Biotechnology Information, https://www.ncbi.nlm.nih.gov/). All the *Salmonella* genomes with annotations of different serotypes were downloaded as of May, 2016, including most of complete genomes as well as draft genomes of type strains or strains of some serovars for which a complete genome was not available. Five subspecies of *S. enterica* (I), *salamae* (II), *arizonae* (IIIa), *diarizonae* (IIIb), and *houtenae* (IV), and a total of 87 serovars of *enterica* (I), were under consideration. MLST (Multilocus Sequence Typing) (Achtman et al., 2012), together with checking the original studies was implemented to identify their serovars. Housekeeping genes, *thrA, purE, sucA, hisD, aroC, hemD*, and *dnaN* were targeted for the MLST scheme (Achtman et al., 2012). The nucleotide sequences were then aligned against seven housekeeping genes. Outcome of sequences were subsequently submitted to the MLST database (http://mlst.warwick.ac.uk/mlst/dbs/Senterica) and assigned existing or novel allele type numbers. The composite sequence STs (sequence types) were defined by the database based on the set of allelic profiles derived from each of the seven loci.

### Determination of homologous groups

OrthoMCL (version 2.0.9) (Li et al., 2003) was used to determine homologous clusters of the pan-genome for subsequent analyses. All extracted protein sequences were adjusted to a prescribed format and were grouped into homologous clusters based on sequence similarity. The BLAST reciprocal best hit algorithm (Moreno-Hagelsieb and Latimer, 2008) was employed with 70% match cutoff, and Markov Cluster Algorithms (MCL) (Enright et al., 2002) were applied with an inflation index of 1.5.

### Phylogenetic analysis

To determine the phylogenetic relationships among *Salmonella* subspecies and different serovars, a phylogenetic tree was inferred from single nucleotide polymorphisms (SNPs) of core genes. According to the identification of homologous clusters, a total of 127,544 genes presented in single-copy orthologous genes shared by all 214 *Salmonella* strains were selected for phylogenetic analysis. For each orthologous cluster, nucleotide sequences were aligned and then concatenated. Custom Perl scripts were used to parse the concatenated sequence and extract SNPs. The organismal phylogeny of SNPs and other genes were inferred using the maximum likelihood (ML) algorithm in RAxML version 8.2.9 (Stamatakis, 2014). The tree reconstruction is repeated 1,000 times, and the percentage of times each interior branch which is noted as bootstrap-value is given. Each tree of genes in the two flagellin gene clusters was constructed by MEGA (Kumar et al., 2016), using Neighbor-Joining method with 1,000 bootstraps.

### Identification of *fliC* and *fljB*

To characterize the evolution pattern of flagellin genes, reference sequences of *fliC, fljB*, and the flanked genes, *fliA, fliB, fliD, fliS, fljA*, and *hin*, were extracted from the *S*. Typhimurium LT2 genome (GenBank accession number AE006468) (McClelland et al., 2001). The relevant homologous clusters in the pan-genome were determined by BLAST with 70% match cutoff and 50% coverage cutoff. In cases of shared homologous genes related to flagellin genes, phylogenetic trees were inferred by ML algorithms in RAxML version 8.2.9 (Stamatakis, 2014). Together with the phylogenetic trees, the flanked gene contents were taken in account to confirm the gene loci of *fliA, fliB, fliC, fliD, fliS, fljA, fljB*, and *hin*.

### Congruence analysis

PAUP version 4.0a152 (Swofford, 1998) was used to conduct maximum-likelihood (ML) analysis of the congruence of gene trees, as described by Feil et al. (2001). The HKY85 model of DNA substitutions was used to estimate α parameter, and the transition/transversion (Ti/Tv) ratio, assuming gamma distribution. The difference between the log likelihood scores of the trees of two genes Δ-lnL was calculated, generating from the comparison of one gene tree against another based on the 99th percentile of the distribution of log likelihood scores for 200 trees from random topology. If the Δ-lnL values for the comparisons is less than that reference gene comparisons within the 99th percentile of this distribution, then these two gene trees should be considered significantly congruent (Octavia and Lan, 2006).

### Regression analysis for evolutionary synchronization

A key element of our study was to determine whether the evolution of flagella gene was associated with the evolutionary rate estimate. To test for this pattern we conducted a least squares linear regression for the genome-wide rate as a function of sampling distance. The MEGA version 7 (Kumar et al., 2016) was used for pairwise distance calculating. A potential shortcoming of this analysis is that the data do not necessarily represent independent samples because some correspond to closely related lineages. This can be addressed by using phylogenetic independent contrasts, or phylogenetic generalized least squares. For the regression of the rate as a function of sampling evolutionary consensus the degree of association was estimated when fitting a regression for a ratio as a function of its denominator.

### Recombination analysis

Potential recombination within *fliB, fliC*, and *fliD* genes was screened using seven methods (RDP, GENECONV, MaxChi, Bootscan, Chimera, SiScan, and 3Seq) implemented in the Recombination Detection Program version 4.88 (RDP4) (Martin et al., 2015). The breakpoints were also defined by RDP4. Similarity between the recombinants and their possible major and minor parents was estimated using BootScan, embedded in RDP4 (Stamatakis, 2014). The recombination event evaluated by RDP4 was considered significant if it satisfied at least two criteria when the *P*-value (*p*) < 0.05 and the RDP recombination consensus score (RDPRCS) was >0.4. When *p* < 0.05 and the RDPRCS was between 0.4 and 0.6, the recombination event was possible. An RDPRCS < 0.4 with *p* < 0.05 indicated the rejection of the recombination event (Martin et al., 2015).

## Results

### Core genome tree revealed detailed phylogenetic relationships among different serovars

**Figure 1:**
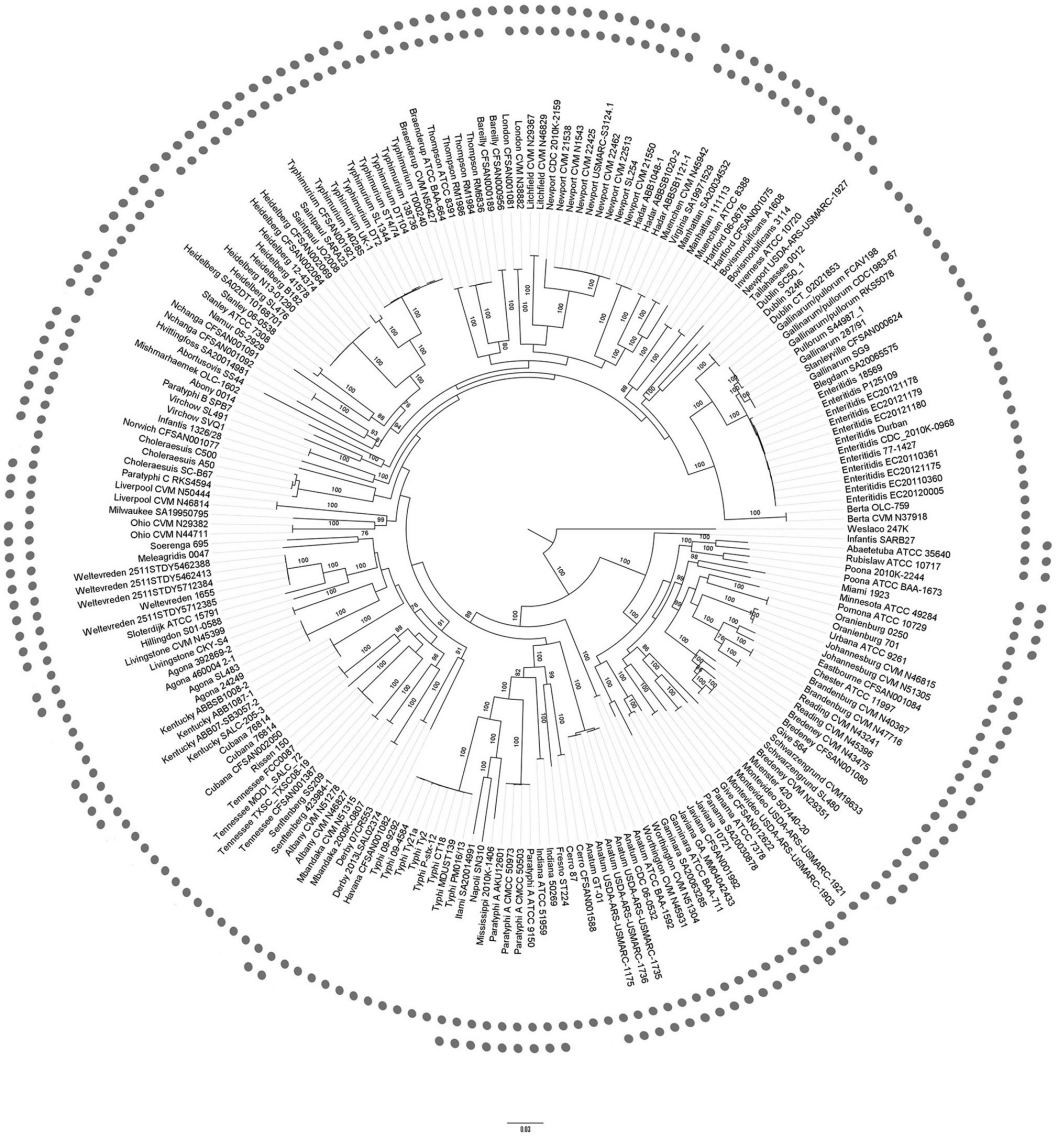
Phylogenetic tree of *Salmonella enterica*. Maximum Likelihood phylogenetic tree based on 57,946 SNPs of 596 single copy core genes of 214 genomes. Bootstrap values (>70%) are presented on the branches. The inner dots indicated the presence of *fliC* gene clusters, while the outer dots indicated *fljB* gene clusters. The bootstrap values near the tips of the trees were omitted. The other four subspecies of *S*. *salamae* (II), *arizonae* (IIIa), *diarizonae* (IIIb), and *houtenae* (IV) were far distant from subspecies *enterica* (I) and omitted.

A total of 214 *Salmonella* genomes were analyzed in this study, consisting of 205 *S. enterica* subsp. *enterica* genomes and 9 genomes of other four *S. enterica* subspecies, which covered 87 serovars. More than one genome (maximum twelve) was included for some famous serovars (e.g. *S*. Enteritidis and *S*. Typhi), while only one genome was considered for most other serovars because no more genomes were available. More detailed information on these genomes was summarized in Table S1. After homologous gene clustering, the total 947,793 proteins were grouped into 14,583 homologous clusters, including common genes represented by 596 single-copy groups of 712 core homologous clusters. A total of 57,946 SNPs in the “core” regions were identified that were present and aligned with high confidence in all taxa. These SNPs were used to construct the phylogeny (Figure 1).

The role of the phylogenetic tree based on SNPs in genome variation and evolution has been documented previously (Filliol et al., 2006; Holt et al., 2008). The core genome tree can support the major genetic group phylogeny and reveal much more phylogenetic detail, including the divergence of major phylogenetic groups. Moreover, the core genome tree also revealed a number of strong concordances with serotyping. From Figure 1, core genome tree was able to categorize *S*. Typhimurium isolates but unable to ultimately separate the *S*. Stanleyville from the *S*. Gallinarum with high resolution. In most cases, the genomes within a cluster belonged to the same serovar, as for *S*. Typhi or Enteritidis. Although some isolates were mixed up with each other, for example, it was hard to differentiate *S*. Give, *S*. Schwarzengrund, and *S*. Bredney, the core genome tree generally separated the serovars from each other.

### The monophasic and biphasic strains were less grouped into the same cluster on core genome tree

The prevalence of phase I and phase II related flagelllin gene locus of the 214 genomes was also indicated in Figure 1. In total, *fliC* gene cluster was found in 207 genomes (96.7%) of 91 serovars (97.8%), including subspecies, while *fljB* gene cluster was found in 138 genomes (64.5%) of 61 serovars (65.6%). The detailed list (Table S2) was dominated by *fliC* and *fljB* genes and included their flanked genes and *hin*. Some well-studied serovars (Enteritidis, Dublin, Typhi etc.) are monophasic the same as the previous study (Selander et al., 1992; Baker et al., 2007). Nevertheless, the *fliC* gene was not found present in the genomes of strain *S*. Poona 2010K-2244, *S*. Heidelberg B182, *S*. Abortusovis SS44, *S. enterica* subsp. *salamae* 3588/07, *S*. Bovismorbificans A1608, *S*. Hadar ABBSB1020-2, and *S*. London CFSAN001081, of which only a draft genome was obtained for these strains. Duplicated annotations and misprediction of some genes that span contig boundaries may occur in draft genomes. The homologs (Table S2) were able to mitigate but not eliminate these limitations. We further checked the contigs and related annotation of seven genomes of *fliC* gene cluster absent, and found some residue of the gene cluster at some contigs' ends in each genome. On the other hand, there was no report on the absence of *fliC* in these serovars, for example, *fliC* should be present in all *S*. Heidelberg (Macnab, 1992). Therefore, the absence of *fliC* gene in these genomes should be a result of misassembly or the related fragment not fully sequenced.

Interestingly, the monophase and biphase being linked to the evolution of *S. enterica* subsp. *enterica* showed discrete phylogenetic clusters on the core genome tree (Figure 1). A few serovars with identical phase, clustered together in the core genome tree. In one larger monophasic clade, with 25 genomes, containing *S*. Dublin, Gallinarum, Pullorum, Stanleyville, Blegdam, Enteritidis, and Berta, all the genomes of each serovar were located together in one distinct cluster in the core genome tree. For the other monophasic serovars, the 45 genomes were identified at different locations, of which another larger clade of 12 genomes, containing *S. enterica* subsp. *enterica* serovars Cubana, Rissen, Tennessee, Senftenberg, and Albany were located adjacent to Derby, Havana, and Agona, but were disrupted by Mbandaka and Kentucky of biphase.

### Evolutionary congruence and phylogenetic analysis implied two distinct evolution patterns of the flagellin gene clusters

Evolutionary analysis of flagellin gene *fliC* and *fljB* in *Salmonella* has been performed previously (Mortimer et al., 2007). However, the co-evolution of these two genes and their flanking genes has never been studied. To evaluate evolutionary congruence between and within the *fliC* and *fljB* clusters, ML analysis was carried out, and the results were summarized in Table 1. As is shown, none of the genes in *fliC* gene cluster were congruent to all the genes in *fljB* gene cluster, and none of the genes in *fljB* gene cluster were congruent to all the genes in *fliC* gene cluster. The gene trees with the largest number of congruencies were those of *fliC* and *hin*, which were congruent to four other gene trees. More concretely, *fliC* gene tree was similar to those of *fliB, fliD, fljA*, and *fljB* gene, and *hin* gene tree was similar to those of *fliB, fliD, fljA*, and *fljB* gene. As for *fliB* (equaling to *fljA* and *fljB* gene trees) and *fljB* (equaling to *fliB* and *fljA* trees), they were congruent to two gene trees. And *fliA* and *fliD* trees were congruent to *fljA* and *fliB* respectively. Overall, only 29.2% (14/48) of the flagellar gene tree comparisons were congruent among these *S*. *enterica* strains. As for the *fliC* and its neighbored genes, *fliB* and *fliD*, they were closely related, while for *fljA* and *fljB* genes, their relationship was more distant. Moreover, there was generally higher level of congruence between *fliC* and *fljB*/*fliD* genes than between *fliA* and *fliB* genes and between *fliD* and *fliS* genes. However, correlation calculations were not available between *fliC* and *fliS* genes, because approximation limit dynamically readjusted too low.

**Table 1:** ML analysis for congruence between each gene tree of the flagella genes analyzed in this study.

	**α^a^**	**Ti/Tv ratio^b^**	**-lnL**	**Δ-lnL (99th)^c^**	***fliA***	***fliB***	***fliC***	***fliD***	***fliS***	***fljA***	***fljB***	***hin***
					Δ**-lnL (99 percentile)**							
*fliA*	0.02	7.59	1,442.96	1,333.38	–	1,446.74	1,855.03	1,588.38	1,336.41	**1,332.98**	1,559.4	1,392.09
*fliB*	0.2	3.37	7,850.6	24,309.07	28,488.81	–	26,254.26	24,838.09	29,386.81	**24,142.47**	**24,295.95**	27,444.31
*fliC*	0.52	1.29	25,042.12	127,229.25	152,120.99	**124,348.02**	–	**125,530.74**	–	**125,113.23**	**124,009.55**	145,249.22
*fliD*	0.29	3.2	7,535.46	35,561.8	43,616.44	**35,031.96**	40,180.51	–	44,377.23	36,789.97	36,477.65	43,028.63
*fliS*	0.01	8.72	877.71	802.22	815.62	850.98	1,059.74	902.58	–	809.76	900.25	842.57
*fljA*	0.15	3.2	1,649.33	2,071.13	2,224.6	2,100.08	2,443.48	2,172.55	2,236.68	–	2,143.27	2,236.19
*fljB*	0.25	2.27	8,965.19	24,268.16	28,402.47	**23,710.94**	25,860.96	24,279.29	28,701.65	**24,179.59**	–	27,639.31
*hin*	0.02	2.67	1,674.34	2,095.84	2,101.85	**1,832.47**	2,264.01	**1,947.05**	2,097.23	**1,795.33**	**1,955.46**	–

a *Nucleotide substitution rate variation between sites, with gamma distribution as the parameter*.

b *Estimated transition/transversion ratio*.

c *Difference in -lnL score from -lnL column (reference data) and the 99th percentile from random topology*.

*^d^ Difference in -lnL score from reference data to each calculated score from other genes. Trees are deemed to be congruent if Δ-lnL is equal to or lower than the 99th percentile of random trees, when compared with reference data (Feil et al., 2001; Octavia and Lan, 2006). Bold character in the table symbolizes congruent tree between these two genes*.

**Figure 2:**
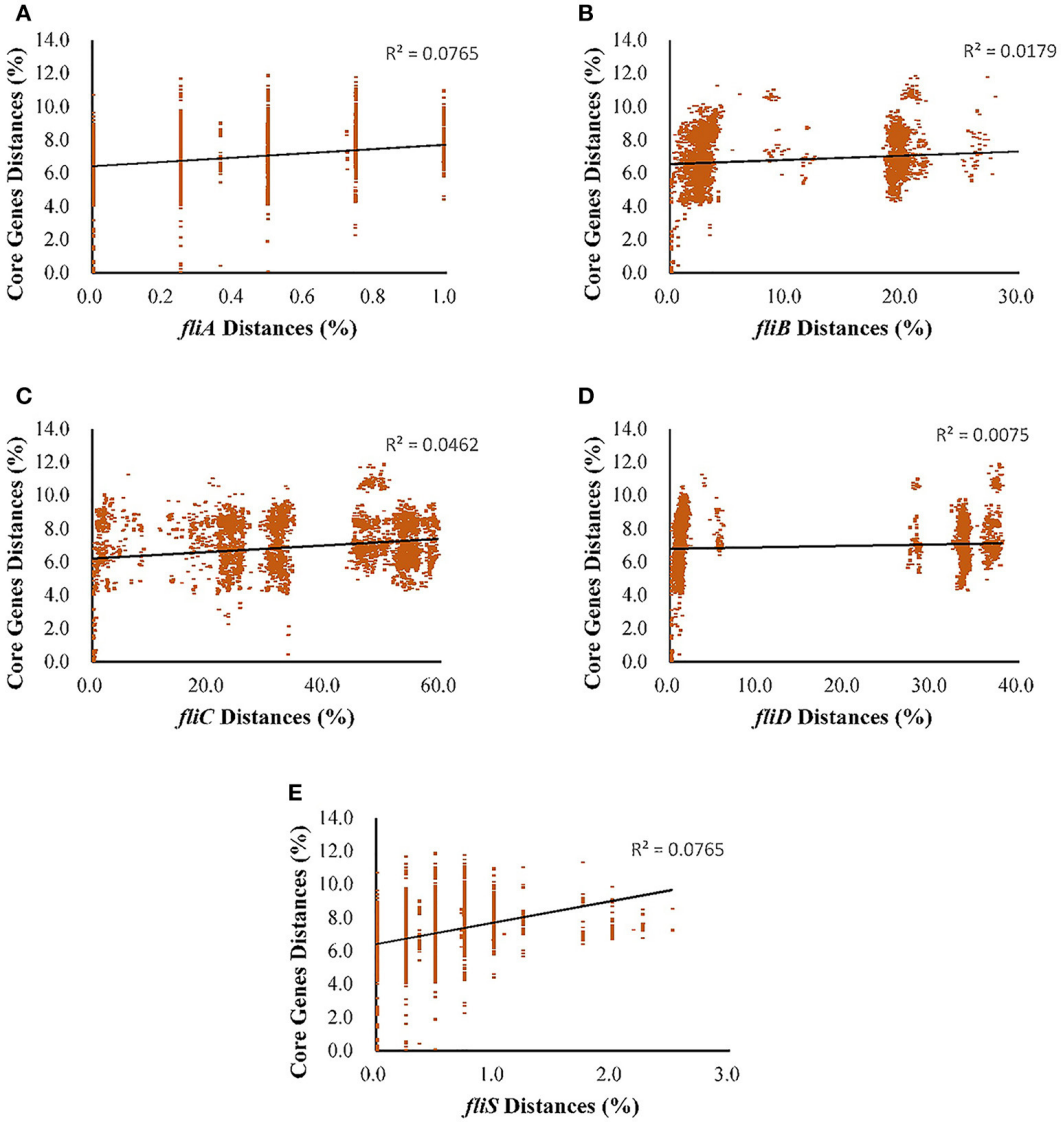
Regressions between each gene of the *fliC* gene clusters and core genetic distance (expected nucleotide substitutions per site) as a function of sampling the evolutionary distance for *S. enterica* (I). **(A)**, *fliA*; **(B)**, *fliB*; **(C)**, *fliC*; **(D)**, *fliD*; and **(E)**, *fliS*. Each dot corresponds to a pair of sampled genomes, and the linear regression is shown as a solid line using least squares; the *R*^2^ coefficients are also shown.

**Figure 3:**
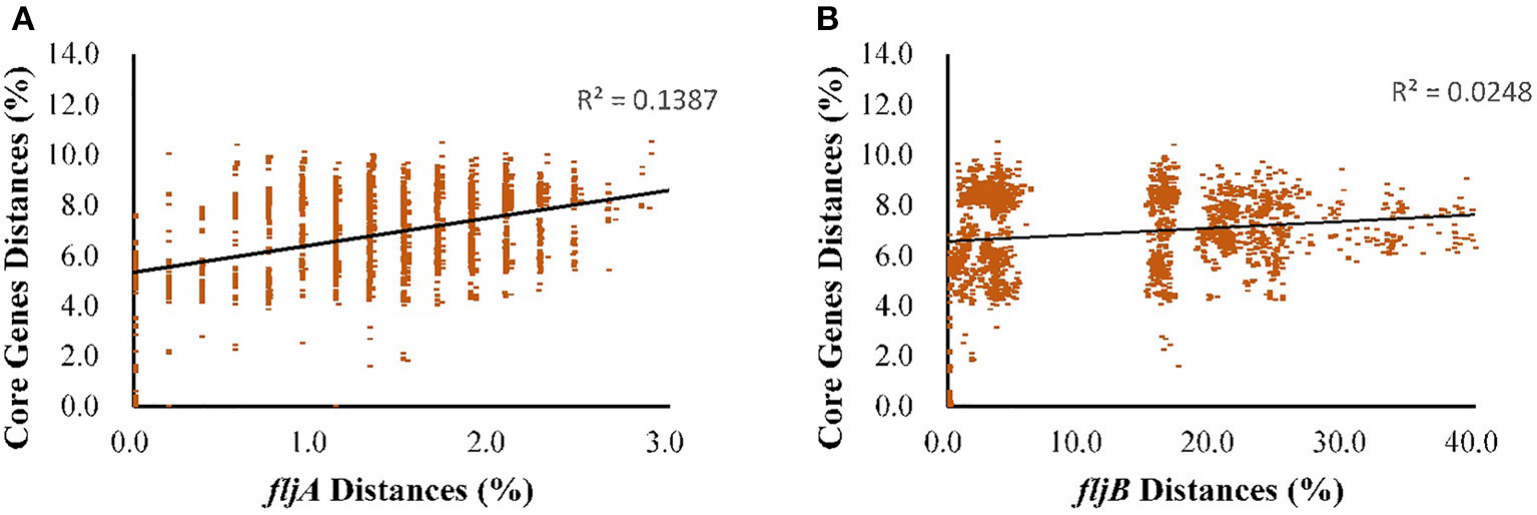
Regressions between each gene of the *fljB* gene clusters and core genetic distance (expected nucleotide substitutions per site) as a function of sampling the evolutionary distance for *S. enterica* (I). **(A)**, *fljA*; and **(B)**, *fljB*. Each dot corresponds to a pair of sampled genomes, and the linear regression is shown as a solid line using least squares; the *R*^2^ coefficients are also shown.

Besides the relationship among the genes in the *fliC* and *fljB* gene clusters, we further studied their congruence to the core genome using linear regression analysis instead of ML analysis, because datasets of the core genome were too large to calculate -lnL score. The mutual information analysis indicated a weak correlation between each flagellin gene and core genome. The low values of both trees for each linear regression indicate a high level of incongruence between individual tree of each gene and the core genome tree, as shown in Figures 2, 3. The topology of *fljB, fliC*, and *fliD* gene trees was much distinct from that of the core genome tree, while those of the *fliA* and *fliS* genes were more similar to the core genome tree than those of *fljB, fliC*, and *fliD* genes. However, the *fljA* tree for serovars displayed a similar topology to that of the core genome tree, while *fljB* did not.

**Figure 4:**
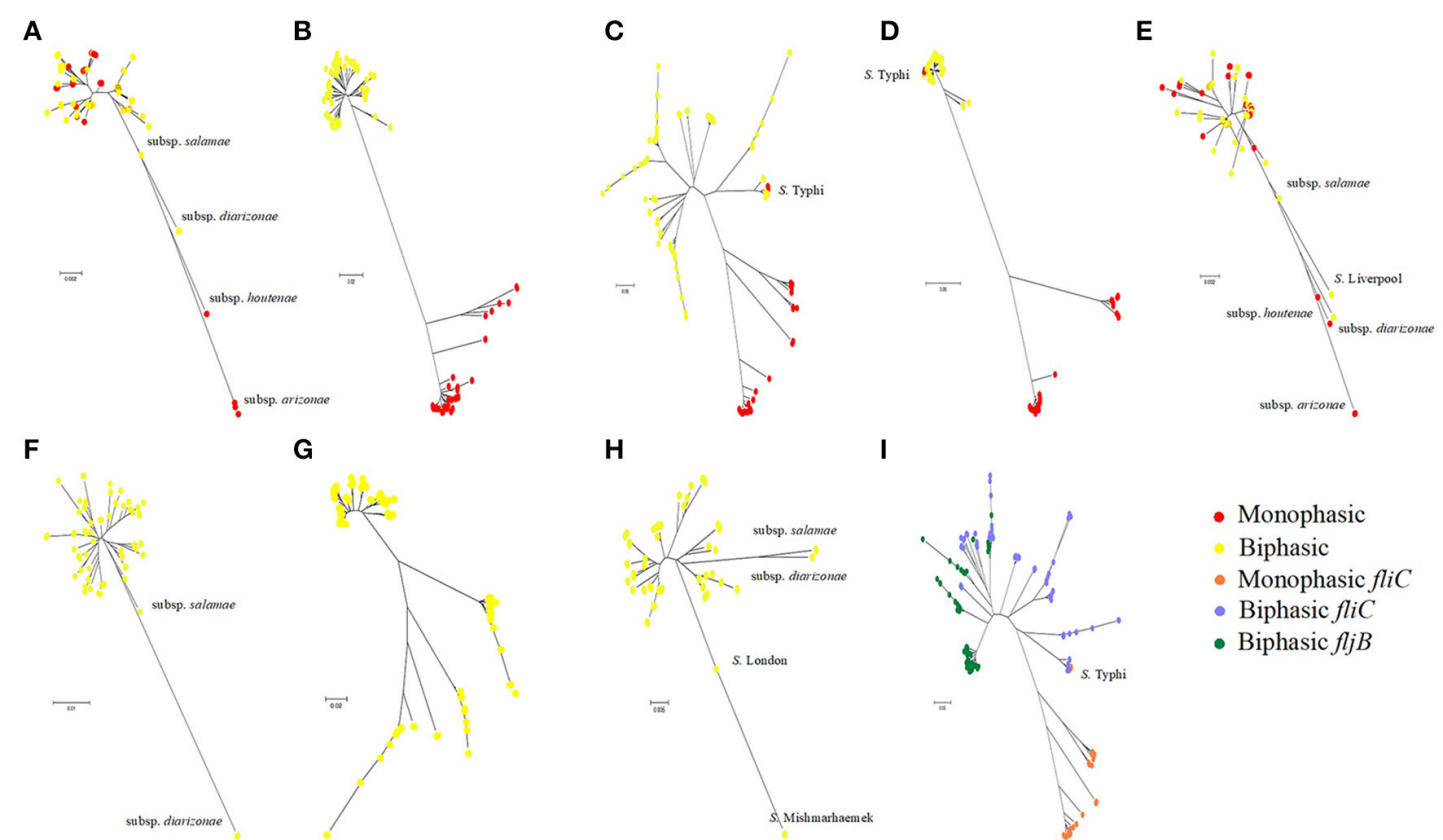
Phylogenetic trees of *Salmonella* flagella related genes. Neighbor-joining phylogenetic trees of flagella related genes are based on the nucleotide sequences: *fliA* **(A)**, *fliB* **(B)**, *fliC* **(C)**, *fliD* **(D)**, *fliS* **(E)**, *fljA* **(F)**, *fljB* **(G)**, *hin* **(H)** and *fliC/fljB* **(I)**. Different color dot implies the monophasic or biphasic serovars.

To infer evolutionary relationships among different serovars, based upon similarities and differences in their genetic characteristics, we constructed phylogenetic trees of *fliA, fliB, fliC, fliD, fliS*, and *fljA, fljB, hin*, and *fliC*/*fljB* (Figure 4). Diagrammatic representation of *fliB, fliC*, and *fliD* indicated bifurcation to form two distinct lineages, where other *Salmonella enteric* subspecies joined together with subspecies enteric serovars. The other four subspecies could be easily recognized from *fliA* and *fliS* gene tree because of their distant branches, which was more consistent with core genome tree. Subspecies *salamae* was close to three neighbors, serovar Weltervreden, Sloterdijk and Kentucky, in *fljB* tree, while these serovars were scattered to different branches in *fljA* tree. The results of phylogenetic trees were consistent to the congruence analysis (Table 1, Figure 2, 3). Dramatically, the individual gene trees of *fliB, fliC* (*fliC* of *S*. Typhi was an exception) and *fliD* were consistent with a clustering dividing monophasic and biphasic serovars. This seemed to imply two distinct evolutionary paths of *fliC*/*fljB* corresponding to monophasic and biphasic serovars.

### Phylogeny of *fliC* and *fljB* showed complex relationships

Because different genes of the flagellin diverge under different evolutionary pressures, we manually curated the phylogenetic pattern of genes in flagellin gene clusters. For all flagellin and their flanked genes, the individual gene trees (Figure 4) can be used to demonstrate the evolutionary story on individual gene basis. To study the genetic evolution of the 207 *fliC* and 138 *fljB* allelic variants, phylogenetic tree based on nucleotide sequences was constructed (Figure 4I). There is a clear separation of monophasic *Salmonella* groups from the biphasic groups based on the *fliC* gene, while all the *fljB* alleles clustered with the biphasic *fliC* alleles. This may either reflect that monophasic were acting as incipient serotypes with a barrier to recombination with biphasic serotypes, or indicate that there were insufficient incidents in which recombination could have taken place between monophasic and biphasic serotypes. However, *S*. Typhi was not clustered into other monophasic genomes as one branch together. Similarly, previous study had also identified that monophasic *S*. Typhimurium existed, of which the serotype was always biphasic (Laorden et al., 2010).

Furthermore, all biphasic *fliC* and *fljB* alleles were analyzed based on the amino acid sequences (Figure S1). The genetic distance between *fliC* gene and *fljB* gene sequences showed no relationship within/among serovars, which was reflected in the phylogenetic tree. For example, serovar Schwarzengrund, Liverpool, and Livingstone were closely related based on *fliC* gene while they were distant based on *fljB*. *S*. Heidelberg and *S*. Newport were clustered together according to the *fljB* gene, while they were clustered separately according to the *fliC* gene. It suggested that the evolutionary histories were independent between *fliC* and *fljB* genes and frequent homologous recombination referring *fliC* and *fljB* occurred between biphasic serovars. The phylogenetic tree of 5′ and 3′ conserved segment of *fliC* and *fljB* genes was also constructed (data not shown), showing the similar division.

### Less recombination events were found between monophasic and biphasic serovars

**Figure 5:**
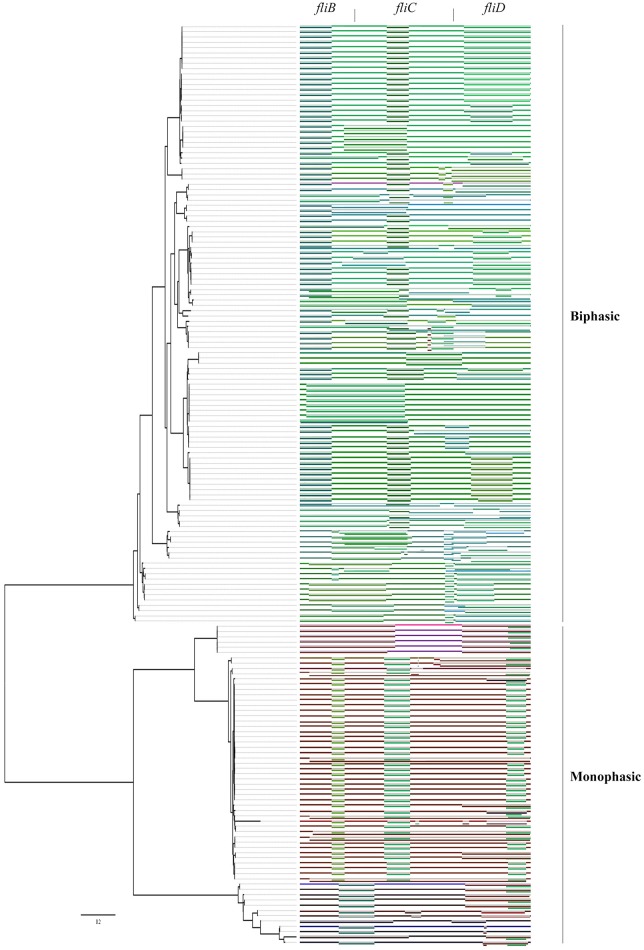
Recombination events in *Salmonella fliBCD* gene cluster. The recombination properties of flagella genes are indicated as different colors on the right, indicating positions of potential recombination events. Piece of sequence from major parent refer to the top of rectangles. Piece of sequence from minor parent is a graphical representation of a sequence fragment that has potentially been derived through recombination from a sequence resembling the next two bars of the rectangle. On the left, a phylogenetic tree of the phase I alleles based on concatenated *fliBCD* nucleotide sequences was constructed by using the maximum-likelihood method with a bootstrap value of 1,000.

Recombination is a significant source of genetic diversity in *Salmonella* (Silverman et al., 1979). This phenomenon, which occurs during co-infection with different strains, is usually observed at the *fliC* and its flanked genes. It is apparent that there were different levels of recombination within flagellar genes of *S. enterica*. Comparison of recombination of monophasic genes with biphasic genes, which represents the unique ability to change flagellar composition, by both ML (Table 1) and recombination detective analysis (Figure 5), showed that recombination occured far more frequently within monophasic or biphasic gene clusters than between them. For example, the serovar Heidelberg, whose major parent (a sequence closely related to that from which the greater part of the recombinant's sequence may have been derived, Martin et al., 2015) was Virchow, and minor parent (a sequence closely related to that from which sequences in the proposed recombinant region may have been derived, Martin et al., 2015) was Newport, did not recombine to any monophasic genes. It was identical to Hartford, whose major parent was Pomona, and minor parent was Anatum. Even though it was found that Senftenberg (monophase) was minor parent for Newport (biphase), and other similar recombinant events of referring small segments were also found, recombination was believed mainly to have occurred within the same phase. Moreover, there were lots of breakpoints and the breakpoints tended to cluster far more around the edges of genes than they did within the central parts of genes (Figure 5).

## Discussion

With the cost of whole genome sequencing (WGS) decreasing dramatically (Didelot et al., 2012) and the technology becoming available for routine use, WGS is very useful and practical in various bacterial infectious studies including diagnostic and public health microbiology (Koser et al., 2012). Many *Salmonella* serovars are human-specific, which may cause gastroenteritis, a self-limiting illness. A few *Salmonella* serovars, especially *S*. Typhi, may elicit typhoid-like diseases and serious systemic infections (Parkhill et al., 2001; Crump and Mintz, 2010). Recent studies support the phylogenetic tree based on SNPs as an outbreak surveillance tool (Allard et al., 2012; Hoffmann et al., 2013; Zhou et al., 2013). The performance of these 214 genomes based on SNPs was validated on the phylogenetic analysis. The fractions of the core genome tree (Figure 1) supported the partition of the taxa in previous study and reflected robust evolutionary relationship of *Salmonella* serovars (Chan et al., 2003; Jacobsen et al., 2011; Zou et al., 2014). Moreover, the rich information of *Salmonella* subspecies *enterica*, covering 87 serovars, provided a framework for determination of the evolutionary relationships of the flagellin alleles to assess the co-evolution and recombination events.

Flagellin genes are considered as a dominant antigen of the adaptive immune response (Haiko and Westerlund-Wikstrom, 2013). The *fliC* gene is present in most of *Salmonella* isolates, and has a homologous locus in *Escherichia coli* (Macnab, 1992). Selection within specific niches might have an impact in antigenic variation (McQuiston et al., 2008). For adapting to environmental changes, *Salmonella* adjust their expression, introducing a second set of antigens, to survive in the new niches. The complex branching division of diphasic serotypes, compared to monophasic serotypes, is suggestive of an expanded mutation rate or recombination in the flagellin when *Salmonella* acquired the second flagellin gene. Perhaps monophasic serotypes obtain enough antigenic diversity from SNPs, letting these serotypes to be successful as monophasic strains (Mortimer et al., 2007). However, the reason why *S*. Typhi is a monophasic serotype and cluster with biphasic serotypes in the phylogenetic trees of *fliBCD* is still unclear. We hypothesize that *S*. Typhi had a trend to be diphasic, and the substitute of *fljB* was present in the history but got lost during speciation.

As an important virulence factor in pathogenic bacteria, especially in *Salmonella* genus, flagellin might regulate their virulence and pathogenesis (Stecher et al., 2004). For example, it is beneficial to immunologic escape by turning on/off the expression of genes related to these structures, niche adaptation by expressing the second flagellin and avoiding predation by different motility or antigenic expression. All of these selective advantages are importantly involved in evolutionary step of having the second flagellin. The gain or loss of their unique ability to encode the major flagellin protein by switching expression can be discussed by an evolutionary model. Based on the phylogenetic tree of the *fliC* and *fljB* genes in the broad serovars involved, it is supposed that the phylogeny of flagellin genes illustrated a strong serovar-specific lineage, with strains of the same serovar clustering either together or in a few distinct branches (Figure 1), as described before (Yue et al., 2015). Clustering of the *fliC* and *fljB* genes showed that *fljB* arose from *fliC* gene duplication (Mortimer et al., 2007), which have highly conserved 5′ and 3′ ends and highly variable center regions (Masten and Joys, 1993; McQuiston et al., 2004). Most studies of flagellin genes focus either on its evolutionary relationships or as a dominant antigen of the sequence diversity (Mortimer et al., 2007) but do not undertake subsequent congruence analysis of their evolutionary process. The systematic approach, ML analysis and regression analysis (Table 1, Figures 2, 3), determined that *fliB, fliC*, and *fliD* gene trees were evolutionarily congruent to each other but not to the core genome. In the regression analysis of *fliA* and *fliS* against the core genome, low levels of correlation were found (low *R*^2^ values). The *fliA* and *fliS* genes were highly conserved within subspecies *enterica* with limited variation (Figure 2), which will disturb the regression analysis, while their phylogenetic trees indicated a clear classification as that of core genome, especially other subspecies (Figure 4). These results suggested that *fliA* and *fliS* evolved congruently with the core genome. Notably, homologous recombination of *fliC* and flanked genes occurred frequently within monophasic and diphasic serovars (Figure 5). These results suggested that the level of clonality of flagellar genes within subspecies I was low.

**Figure 6:**
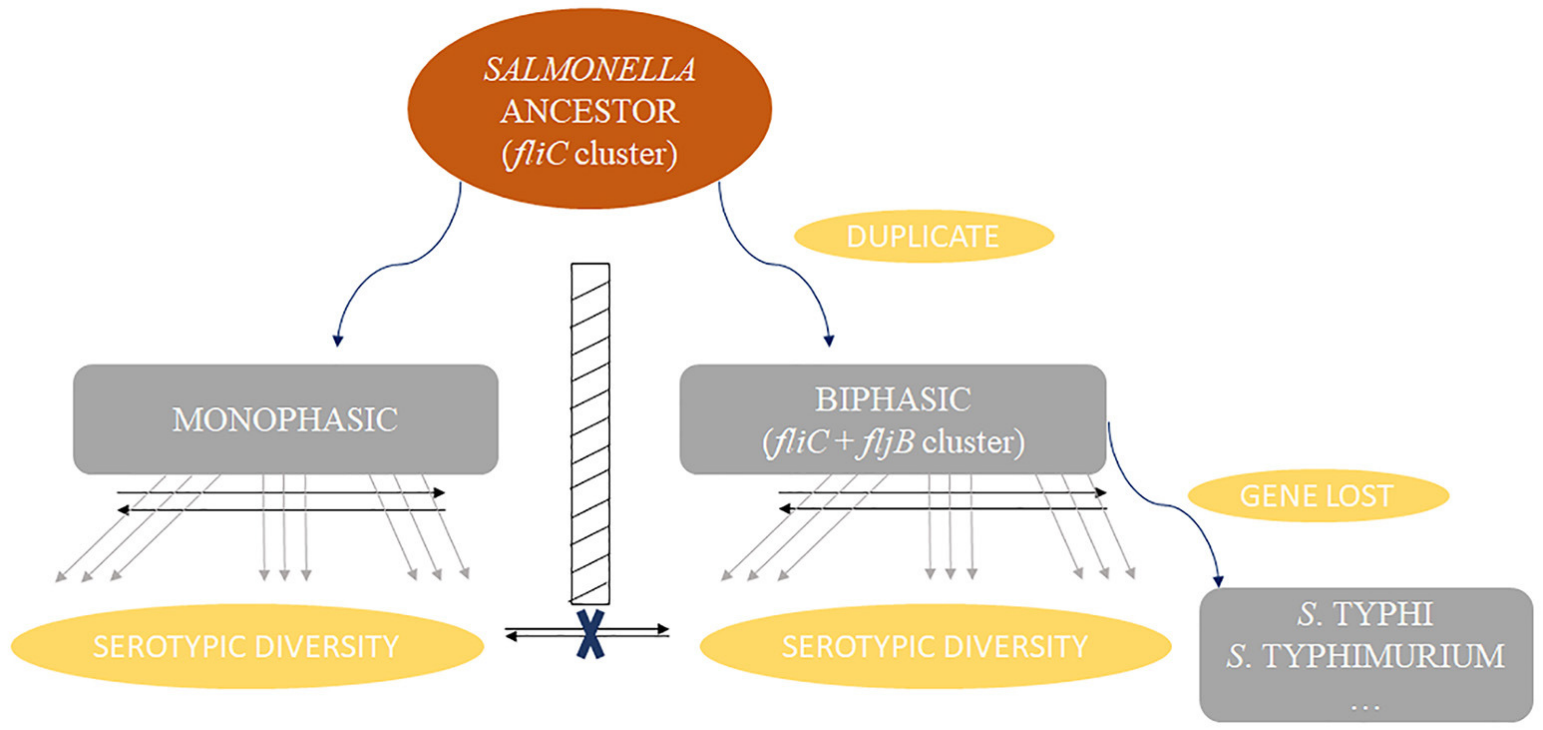
Schema diagram showing the two distinct evolution patterns of *Salmonella* fagellin gene clusters. Homologous recombination (represented by black arrows) rarely occurred between monophasic and biphasic serovars, but frequently occurred within the same phase. Some serovars and some isolates of certain serovars should have been biphasic historically but become monophasic because of gene loss of *fljB* gene cluster, such as serovar Typhi and Typhimurium.

Dramatically, the phylogenetic trees of *fliB, fliC*, and *fliD* revealed a similar division of the serovars into two distinct groups, corresponding to monophasic and biphasic serovars, respectively. This implied two distinct evolutionary patterns of *Salmonella fliC*/*fljB* gene cluster. This division was not pointed out previously, but similar phylogenetic structures of *fliC*/*fljB* were reported (Smith et al., 1990; Mortimer et al., 2007). It was divided into Non-G antigens and G antigens based on *fliC*_g, m, s_ encoding three factors. Through the phylogenetic tree in Mortimer (Mortimer et al., 2007) study, Non-G group and G group were clustered into different branches, which equaled to biphasic and monophasic groups respectively. According to these results, a flow diagram was constructed to demonstrate the evolutionary path of the monophasic and biphasic gene clusters (Figure 6). The *Salmonella* ancestor should just harbor a *fliC* gene cluster as most of *E. coli* isolates (McQuiston et al., 2008). Subsequently, some lineages suffered *fliC* duplication to assemble an *fljB* gene cluster under certain natural selection conditions (biphasic), while the others not (monophasic). Both monophasic and biphasic lineages evolved and adapted to different environments and occupied diverse ecological niches, resulting in H antigen (serovar) diversification. Ancestors of some biphasic serovars might have lost *fljB* gene cluster under natural selection because it was useless, becoming monophasic again (e g., *S*. Typhi and some *S*. Typhimurium isolates). Frequent homologous recombination occurred within monophasic and biphasic serovars. Finally, the current diversity of *Salmonella* was formed. This theory assumed that *fljB* gene cluster should perform a role together with *fliC* cluster in most biphasic serovars, but present knowledge suggested that just one of them was expressed in a cell (Imre et al., 2005). Therefore, the role of *fljB* needs to be further determined to clarify the implication of the two distinct evolutionary patterns.

Collectively, we provide the viewpoint that flagellin genes of *S. enterica* subsp. *enterica* represents two evolutionary patterns with a barrier between monophasic and biphasic serovars. The evolutionary barrier may inhibit an overlapping recombination. However, it is still unclear what the implication of the barriers on the immunological escape, niche adaptation, and avoiding predation is. That is an interesting area for further study. Besides, different serovar genome repertoires were included, suggesting that the core genome evolution of *S. enterica* was accompanied by *fliA*/*fliS* but distinct from those of *fliBCD* and *fljB* gene cluster. This study will provide a robust genetic framework for future studies on the evolution of the phase I and phase II flagellar characteristics in *S. enterica*.

## Author contributions

YL and DFZ designed and initiated the study, interpreted the results, and wrote the manuscript; YL downloaded and analyzed the data; LZ, XZ, LX, and XS contributed to discussion of the results and improvement of the manuscript and designed the outline of this study and manuscript and provided laboratory equipment and space.

### Conflict of interest statement

The authors declare that the research was conducted in the absence of any commercial or financial relationships that could be construed as a potential conflict of interest.

## Supplementary material

Table S1 Complete list of all *Salmonella* genome sequences. The 214 genomes included 205 *Salmonella enterica* subsp. *enterica* and nine genomes of four other subspecies, *arizonae, diarizonae, houtenae*, and *salamae*.

Table S2 Presence/absence of flagellin genes of all analyzed *Salmonella*. The *fliA, fliB, fliC, fliD*, and *fliS* for *fliC* clusters, and *fljA, fljB*, and *hin* for *fljB* clusters were analyzed. a –, absence of the gene; gene name, the locus tag in the genome.

Figure S1 The genetic relationships of biphasic *fliC* and *fljB* genes. Maximum Likelihood phylogenetic tree shows the genetic relationships of biphasic *fliC* and *fljB* genes. It shows the details of the biphasic lineage in Figure 4I. The *fliC* and *fljB* genes were shown in green and blue characters, respectively. Each square represented a group of alleles with identical amino acid (83 sequence types in total). The *fliC* and *fljB* genes from the same genomes were linked by solid lines.
