# Supplementary figures and images for: Comprehensive Analysis Reveals Two Distinct Evolution Patterns of Salmonella Flagellin Gene Clusters
Source: Front Microbiol. 2017 Dec 22;8:2604. doi: 10.3389/fmicb.2017.02604 (PMC5744181; doi:10.3389/fmicb.2017.02604)

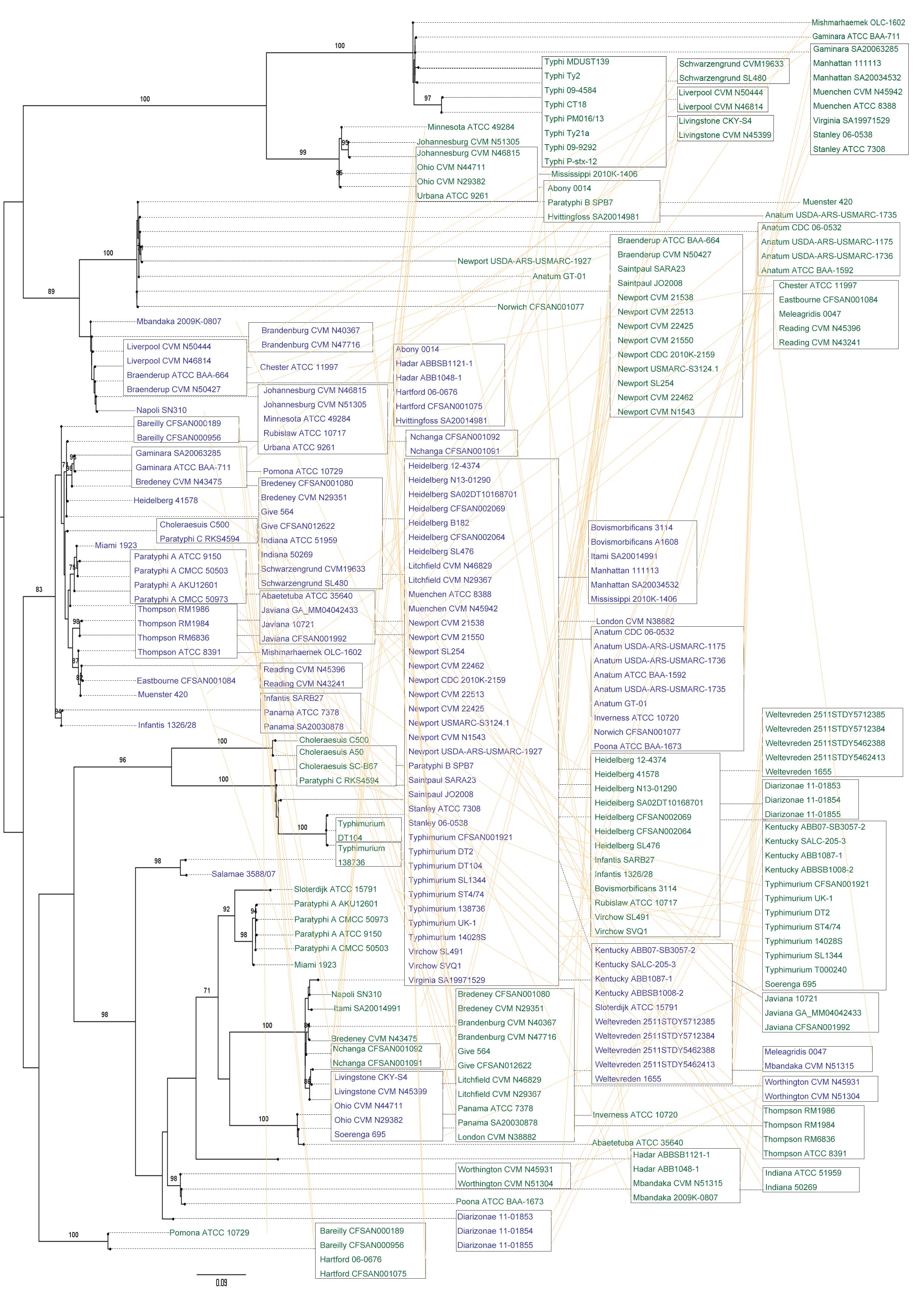

Supplement: Figure S1 — The genetic relationships of biphasic fliC and fljB genes. Maximum Likelihood phylogenetic tree shows the genetic relationships of biphasic fliC and fljB genes. It shows the details of the biphasic lineage in Figure 4I. The fliC and fljB genes were shown in green and blue characters, respectively. Each square represented a group of alleles with identical amino acid (83 sequence types in total). The fliC and fljB genes from the same genomes were linked by solid lines. [file Image1.JPEG]
